# Methylation Status of Alu and LINE-1 Interspersed Repetitive Sequences in Behcet's Disease Patients

**DOI:** 10.1155/2016/1393089

**Published:** 2016-03-30

**Authors:** Şahru Yüksel, Selma Ozbek Kucukazman, Gülten Sungur Karataş, Mehmet Akif Ozturk, Sasiprapa Prombhul, Nattiya Hirankarn

**Affiliations:** ^1^Center of Excellence in Immunology and Immune-Mediated Diseases, Department of Microbiology, Faculty of Medicine, Chulalongkorn University, Bangkok 10330, Thailand; ^2^Ankara Education and Research Hospital, Altindag, 06340 Ankara, Turkey; ^3^Gazi University Faculty of Medicine, Yenimahalle, 06560 Ankara, Turkey

## Abstract

Behcet's Disease (BD) is a multisystem chronic inflammatory disease. The pathology is believed to involve both genetic susceptibility and environmental factors. Hypomethylation leading to activation of interspersed repetitive sequences (IRSs) such as LINE-1 and Alu contributes to the pathologies of autoimmune diseases and cancer. Herein, the epigenetic changes of IRSs in BD were evaluated using combined bisulfite restriction analysis-interspersed repetitive sequences (COBRA-IRS). DNA from neutrophils and peripheral blood mononuclear cells (PBMCs) of BD patients with ocular involvement that were in active or inactive states and healthy controls were used to analyze LINE-1 and Alu methylation levels. For Alu sequences, significant differences were observed in the frequency of ^u^C^u^C alleles between PBMCs of patients and controls (*p* = 0.03), and between inactive patients and controls (*p* = 0.03). For neutrophils, the frequency of ^u^C^u^C was significantly higher between patients and controls (*p* = 0.006) and between inactive patients and controls (*p* = 0.002). The partial methylation (^u^C^m^C + ^m^C^u^C) frequencies of Alu between inactive patients and control samples also differed (*p* = 0.02). No statistically significant differences for LINE-1 were detected. Thus, changes in the methylation level of IRS elements might contribute to the pathogenesis of BD. The role of Alu transcripts in BD should be investigated further.

## 1. Introduction

Behcet's Disease (BD) is a complex systemic inflammatory disorder, generally characterized by recurrent oral aphthous ulcers, genital ulcers, and uveitis [1]. However the clinical spectrum is wide and the manifestations of the disease, such as the involvement of the nervous and gastrointestinal systems, and vasculitis in large veins and arteries vary considerably depending on gender, individual differences, and ethnicity and can lead to mortality and organ loss in severe cases.

BD shares many similarities with autoinflammatory diseases, which comprise a group of disorders caused by genetic mutations in the components of the innate immune system. Among these similarities are the nonspecific inflammatory response, which manifests itself as flares and remissions, with the main involvement of neutrophils, and clinical findings, such as fever, increased acute phase proteins, and overexpression of proinflammatory cytokines, such as IL-1 B and TNF-alpha during the attacks [2–5]. Gene mutations in Familial Mediterranean fever, a prototypical autoinflammatory disease, have been found frequently in BD patients and are suggested to contribute to the severity of the disease [6]. BD also shows critical differences from classical autoimmune diseases, such as male dominance in severe disease [7–9], lack of association with autoimmune HLA class II haplotypes, and, more importantly, absence of disease-specific high titer autoantibodies or antigen-specific T cells [10].

The etiology of the disease is unknown; however, both genetic and environmental factors have been implicated in its pathogenesis. Occasional familial occurrence [11], genetic distribution along the ancient Silk Road, and an association with HLA-B51 are some of the factors pointing toward genetic involvement [12, 13]. Environmental conditions, such as bacterial or viral infections, are thought to trigger the disease in genetically susceptible individuals [10]. To date, HLA B-51 shows the strongest association with BD but accounts for less than 20% of the risk [14], which suggests the involvement of other genetic factors. Genome wide association studies revealed other candidate genes, such as* IL-10, IL23R, STAT4, CCR1,* and* KLRC4,* that could contribute to BD pathogenesis [15, 16]. One genome wide association study also suggested the epistasis between HLA-B51 and ERAP1 gene [17]. In another study, copy number variation in the* DEFA1* defensin gene was associated with susceptibility to intestinal involvement in BD [18]. Other recent studies reported that more candidate gene polymorphisms involved in BD included* ATG5, FAS,* pre-miR-196a2, miR-182, and miR-146a [19–23]. In 2014, a genome wide methylation array study in monocytes and CD4+ T lymphocytes revealed the role of epigenetics in BD pathogenesis. The authors identified abundant aberrant methylation patterns of cytoskeletal element genes in monocytes and CD4+ T lymphocytes as a major contributor to disease pathogenesis [24]. Importantly, it was reported that, after treatment, when the patients were in remission, their methylation patterns reversed back to the patterns seen in healthy controls, suggesting that a better understanding of epigenetic alterations might help us to find new disease markers and treatment options for BD patients with different symptoms. Active transcription factors and specific proteins that affect the binding of methyltransferases in BD likely determine the specific genes that are hypomethylated. However, it is clear that the regulation of methylation is defective in BD.

Almost half of the human genome comprises interspersed repetitive sequences (IRSs) [25], which can be divided into DNA transposons and retroelements. There are two types of retroelements, non-long terminal repeats (LTRs) and LTRs; non-LTR retroelements are further classified into long interspersed nuclear element (LINE) retroposons, which are represented by LINE-1 (20.1%), and short interspersed nuclear elements (SINEs), which are primarily represented by Alu sequences (13.1%) [26]. Most IRS sequences are densely methylated in normal somatic cells. In this state, they are mostly inactive and remain silent. However, hypomethylation and reactivation of these sequences are suggested to have several functional roles, such as controlling the activity of genes by regulating enhancers and repressors, or acting as an alternative promoter upon mobilization, which could lead to insertional mutations and chromosomal instability.

There is growing evidence for the contributions of epigenetic alterations to the pathogenesis of cancer and autoimmune diseases [27]. Recently, hypomethylation and reactivation of IRSs were shown to be important in autoimmune disorders such as systemic lupus erythematosus (SLE), rheumatoid arthritis (RA), and psoriasis. In SLE, LINE-1, but not Alu, was shown to be significantly hypomethylated in CD4+ T lymphocytes and neutrophils of active patients compared with inactive patients and healthy controls [28, 29]. Similarly, significant hypomethylation of LINE-1, but not Alu, in the epidermis of psoriasis patients was reported [30]. In RA, abundant LINE-1 transcripts were detected in synovial fluids from patients [31], as well as hypomethylation in LINE-1 promoters in synovial fibroblasts [32].

In this report, we investigated for the first time the methylation in LINE-1 and Alu repetitive sequences from active and inactive Behcet's patients compared with healthy controls.

## 2. Materials and Methods

### 2.1. Patients and Controls

Patients who fulfilled the International Study Group criteria for Behcet's Disease were recruited from the ophthalmology clinic of the Ankara Education and Research Hospital. BD patients with an ongoing severe ocular involvement (uveitis, retinitis, papillitis, or vasculitis) were defined as the active group (*n* = 12; eight male, four female). BD patients that were free of any active ocular involvement for at least 3 months (confirmed by microscopic and imaging findings) and any extraocular involvement for at least 4 weeks were considered as the inactive group (*n* = 17; 12 men, five women). Fifteen ethnically matched healthy volunteers who were free of any acute or chronic immune-mediated illness (chronic infection, allergic and autoimmune diseases) at the time of sample collection were used as the control group (*n* = 15; eight women, seven men). None of the healthy controls has family history of BD and any immune-mediated diseases. The average and age range of the patients with active BD and inactive BD and healthy controls were 32.9 ± 9.0 (21–57), 36.8 ± 9.7 (18–49), and 33.8 ± 7.9 (20–48) years, respectively (mean ± SD). The details of the patient group are shown in Table 1. This study was approved by the Bogazici University Institutional Review Board for Research with Human Subjects. Informed consent was obtained from all patients and control subjects before entering the study.

### 2.2. Neutrophil and PBMC Isolation

Blood samples were collected into heparinized-Vacutainer tubes (Becton, Dickinson and Company, Plymouth, UK) and cell isolation was performed using density gradient centrifugation. In brief, the collected blood was carefully layered on top of lymphoprep (http://AXIS-SHIELD-PoC.com/, Oslo, Norway) at a blood : lymphoprep ratio of 2 : 1. The peripheral blood mononuclear cell (PBMC) layer was collected and washed once with Roswell Park Memorial Institute (RPMI) medium and stored at −80°C for DNA isolation. Remaining polymorphonuclear granulocytes (PMNs) or neutrophils were diluted 1 : 1 with RPMI and layered carefully on top of polymorphprep (http://AXIS-SHIELD-PoC.com/, Oslo, Norway) at a 1 : 1 ratio. The PMN fraction obtained from the gradient centrifugation was collected and washed once with RPMI. The red blood cells were removed from the PMN fraction using hypotonic lysis buffer. PMNs were collected and centrifuged and the pellet was stored at −80°C for further experiments. The purity of the PMNs was confirmed by Giemsa-Wright staining.

### 2.3. Combined Bisulfite Restriction Analyses (COBRA)

DNA was extracted from collected cells using a Qiagen EZ DNA isolation kit (Qiagen, Valencia, CA, USA), according to the manufacturer's protocol. An EZ DNA methylation kit (Zymo Research, Orange, CA, USA) was used for the bisulfite conversion of 500 ng of the DNA, following the protocol supplied by the manufacturer. Global assessment of methylation levels in LINE-1 and Alu sequences was performed using the combined bisulfite restriction analysis-interspersed repetitive sequences (COBRA-IRS) technique, as previously described [28–30] This technique enabled us to detect methylation levels in two CpG sites in LINE-1 and Alu repetitive elements among thousands of copies present in the human genome. The following primers were used for amplification of the bisulfite treated DNA: Alu forward 5′ GGY GYG GTG GTT TAY GTT TGT AA 3′ and Alu reverse, 5′ CTAACTTTTTATATTTTTAATAAAA ACRAAATTTCACCA 3′; LINE-1 forward 5′ GTTAAAGAAAGGGGTGAYGGT 3′ and LINE-1 reverse 5′ AATACRCCRTTTC TTAAA CCRATCTA-3′. The 92 bp PCR product for LINE-1 was obtained using the following cycle conditions: initial denaturation at 95°C for 5 min; 35 cycles of 95°C for 30 s, 55°C for 30 s, 72°C for 45 s; and a final extension at 72°C for 7 min. The 133 bp Alu PCR product was obtained by using the following cycle conditions: initial denaturation at 95°C for 5 min; 40 cycles of 95°C for 30 s, 57°C for 30 s, and 72°C for 45 s; and a final extension at 72°C for 7 min. After amplification, the LINE-1 PCR products were digested with two units of* Taq*I (Fermentas International, Canada) and eight units of* Tas*I (Fermentas International) restriction enzymes. The Alu PCR products were digested with four units of* Taq*I. Each digestion reaction was incubated overnight at 65°C and separation was performed the following day on 8% Tris-borate-EDTA (TBE) polyacrylamide gels. The gel was stained with SYBR green nucleic acid stain (Invitrogen, Carlsbad, CA, USA). The intensity of the staining of the DNA fragments was measured using a phosphorimager equipped with ImageQuant software (Molecular Dynamics, GE Healthcare, Slough, UK). The samples were normalized against the data obtained from DNA samples of HeLa, Jurkat, and Daudi cell lines to eliminate interassay variation between each experiment.

### 2.4. LINE-1 Methylation Analysis

**Figure 1:**
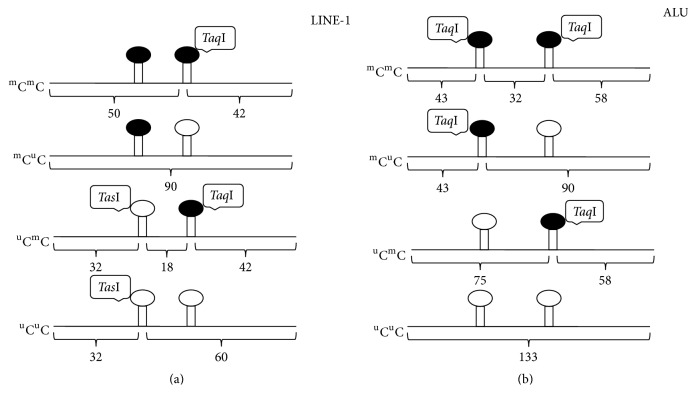
LINE-1 and Alu methylation analysis using COBRA. Restriction enzyme* Taq*I cuts after methylated cytosine residues (represented by closed circles) and enzyme* Tas*I cuts after unmethylated cytosine residues (represented by open circles). (a) Bisulfite treated DNA was amplified to generate LINE-1 amplicons and then digested with* Taq*I and* Tas*I enzymes to obtain fragments representing the four possible methylation patterns (^m^C^u^C, ^u^C^u^C, ^m^C^m^C, and ^u^C^m^C), producing bands at 92, 60, 50, and 18 bp, respectively. (b) Bisulfite treated DNA was amplified to generate Alu amplicons and then digested with* Taq*I to obtain fragments representing the four possible methylation patterns (^u^C^u^C, ^m^C^u^C, ^u^C^m^C, and ^m^C^m^C), producing bands at 133, 90, 75, and 32 bp, respectively.

The intensity of the fragments generated by COBRA from LINE-1 depended on the methylation state of the two CpG dinucleotides and were represented by the following unique bands: fully methylated (^m^C^m^C) 50 bp; partial methylation (^u^C^m^C) 18 bp and (^m^C^u^C) 92 bp; and hypomethylation (^u^C^u^C) 60 bp (Figure 1(a)). To calculate the percentage methylation for each of the four possible states, initially, the intensity of each band was divided by the length (bp) of the double-stranded DNA: *A* = intensity of the 92 bp fragment/92, *B* = intensity of the 60 bp fragment/56, *C* = intensity of the 50 fragment/48, *D* = intensity of the 42 bp fragment/40, *E* = intensity of the 32 bp fragment/28, and *F* = [(*D* + *E*) − (*B* − *C*)]/2. Note that the length of the double-stranded DNA was calculated due to the overhanging end from restriction enzymes. In addition, please also note that since we could not see 18 bp band clearly, we calculate intensity of 18 bp using *F* instead. The values obtained were then applied to the following formula; %  ^m^C^m^C = 100 × *C*/(*A* + *B* + *C* + *F*), %  ^u^C^m^C = 100 × *F*/(*A* + *B* + *C* + *F*), %  ^m^C^u^C = 100 × *A*/(*A* + *B* + *C* + *F*), and %  ^u^C^u^C = 100 × *B*/(*A* + *B* + *C* + *F*), % Methylation (%  ^m^C) = (^m^C/(^m^C + ^u^C)) = 100 × (*A* + 2*C* + *F*)/(2*A* + 2*B* + 2*C* + 2*F*).

### 2.5. Alu Element Methylation Analysis

The COBRA method used in this study is based on the detection of methylation status of two CpG dinucleotides in the 133 bp Alu amplicon by the restriction enzyme* Taq*I, as shown in Figure 1(b). The different methylation states of these two CpG sites were analyzed in four groups. Fully methylated loci (^m^C^m^C), represented by a 32 bp fragment; unmethylated loci (^u^C^u^C) represented by a 133 bp fragment; and partially methylated loci, (^m^C^u^C), represented by a 90 bp fragment, and (^u^C^m^C), represented by a 75 bp fragment (Figure 1(b)).

Enzymatic digestion of 133 bp COBRA-Alu products with the* Taq*I enzyme generated bands of 133, 90, 75, 58, 43, and 32 bp in length, with different intensities based on the methylation status of the two CpG dinucleotides. The frequency of each pattern was calculated according to the following formulas. First, the intensity of each band was divided by the length (bp) of the double-stranded DNA: *A* = intensity of 133 bp fragment/131, *B* = intensity of 58 bp fragment/56, *C* = intensity of 75 bp fragment/73, *D* = intensity of 90 bp fragment/88, *E* = intensity of 43 bp fragment/41, and *F* = intensity of 32 bp fragment/30. Next, the percentage of hypermethylated loci (^m^C^m^C) = 100 × *F*/(*A* + *C* + *D* + *F*), the percentage of partially methylated loci (^u^C^m^C) = 100 × *C*/(*A* + *C* + *D* + *F*), (^m^C^u^C) = 100 × *D*/(*A* + *C* + *D* + *F*), and the percentage of hypomethylated loci (^u^C^u^C) = 100 × *A*/(*A* + *C* + *D* + *F*), the percentage of methylated loci, and % Methylation (^m^C) = (^m^C/(^m^C + ^u^C)) = 100 × (2*F* + *D* + *C*)/(2*A* + 2*C* + 2*D* + 2*F*) were calculated.

### 2.6. Statistical Analyses

Independent sample *t*-tests (two-tailed) were applied to compare various methylation patterns of LINE-1 and Alu among active, inactive, and healthy controls. In some groups where the data were not in normal distribution, we used Mann-Whitney test instead. A *p* value of <0.05 was considered statistically significant. Calculations were performed using GraphPad v5.0 (San Diego, CA) and SPSS software version 15.0 (SPSS Inc., Chicago, IL).

## 3. Results

### 3.1. LINE-1 Methylation Analysis

The differences in the frequency of methylation in genomic DNA obtained from PBMCs and neutrophils of patients with active and inactive BD as well as the controls were compared. The averages of the frequency of methylation are shown in Table 2. There were no statistically significant differences in the overall methylation between BD patients and healthy controls or between the active and inactive groups in both PBMC and neutrophil subsets. When we compared the differences between each methylation pattern (i.e., ^m^C^m^C, ^u^C^u^C, ^u^C^m^C, and ^m^C^u^C), we also found no significant differences among the groups in the cell types.

### 3.2. Alu Methylation Analysis

The frequency of Alu methylation and paired comparisons in the PBMC and neutrophil cell types are shown in Table 3. There were no statistically significant differences in overall methylation levels of PBMCs of active patients when compared with controls and inactive patients, as well as between controls and inactive patients. However, the frequency of ^u^C^u^C in PBMCs was significantly higher in BD patients compared with controls (*p* = 0.03), particularly for the inactive patients group compared with the controls (*p* = 0.03). In the neutrophil population, no statistically significant differences were observed between the groups in terms of the overall methylation. However, the level of ^u^C^u^C was higher, indicating a hypomethylated status of Alu sequences in neutrophils of BD patients compared with those of healthy controls (*p* = 0.006). This finding was more striking in the inactive group compared with controls (*p* = 0.002). When we analyzed the ^u^C^m^C + ^m^C^u^C pattern, a significant difference was also found between inactive patients and controls (*p* = 0.02).

## 4. Discussion

Hypomethylation of IRSs has been investigated extensively and shown to have a role in pathogenesis of complex diseases such as cancer and autoimmune related conditions [27, 33, 34]. The mechanism of how IRS hypomethylation contributes to the pathogenesis of autoimmune diseases is unclear; however, it has been suggested that it could cause aberrant regulation of neighboring genes that have a role in the regulation of the immune response [35, 36], as well as causing nonspecific autoimmune activation via the presence of viral dsRNA or ssRNA transcripts in the circulation [37–39]. In this study, we investigated the role of global hypomethylation in BD and found that levels of overall methylation were not statistically different for Alu and LINE-1 among the active, inactive, and healthy control groups. However, we observed statistically significant differences in the frequency of ^u^C^u^C and partial methylation of Alu in BD compared with healthy controls in both PBMCs and neutrophils. This hypomethylation status was more significant in inactive patients compared with healthy controls. Interestingly, this finding is quite different from our previous finding in SLE [28, 29].

The mechanisms driving demethylation of IRSs are complex and not fully understood; however, age-dependent and age-independent mechanisms accounting for the hypomethylation of IRSs have been reported. Both age-related (possibly caused by malnutrition and/or oxidative stress) and unrelated loss of methylation events have been proposed to occur in Alu elements, whereas hypomethylation of LINE-1 sequences was reported to be age-independent [40, 41]. LINE-1, but not Alu, hypomethylation was reported in SLE and several other autoimmune diseases. Contrastingly, for many different types of cancer, both Alu and LINE-1 hypomethylation play a role in carcinogenesis [33, 34]. In particular, LINE-1 methylation has been suggested as a poor prognostic factor for several cancer types [42]. In this study, we observed a significant increase in the frequency of the ^u^C^u^C allele in both PBMCs and neutrophils in BD patients compared with controls. In addition, the frequency of partial methylation was higher in the controls compared with inactive patients in DNA obtained from neutrophils.

Although earlier reports proposed that Alu hypomethylation is age-related [40], the decrease was only significant for the 34–68-year age interval. The average ages of our control and patient cohort were comparable; therefore, the changes detected are unlikely to be related to the aging process. These findings indicated that there might be an increased tendency for hypomethylation of Alu sequences in BD patients. At present, it is unclear why there should be more change between the inactive group and the controls; however, previously, hypomethylation was found in inactive cells that represented a primed stage, suggesting that the cells were more ready to respond [29].

Interestingly, a recent report observed that the accumulation of Alu transcripts in retinal-pigmented epithelial cells induced NLRP3 activation through mitochondrial oxygen species generation, resulting in production of the proinflammatory cytokine IL-18, which contributed to the pathogenesis of age-related macular degeneration (AMD) [43]. Recently, another study showed that iron accumulation is responsible for Alu transcript accumulation and contributed to the inflammatory phenotype in AMD [44]. Thus, Alu transcript overexpression might induce inflammatory phenotypes in other diseases with similar etiologies. The BD patients who participated in this study were admitted to the eye clinic with severe eye complications, such as uveitis, in addition to oral and urogenital ulcers. An earlier report suggested that at low concentrations Alu transcripts were activators of dsRNA-dependent protein kinase R, which has an important role in the antiviral response, such as the activation of NF-KB, one of the key molecules in the initiation of the inflammatory response. It is not clear whether the slight hypomethylation of Alu seen in BD patients has any effect on the pathogenesis of BD. It should be kept in mind that BD is complex and manifests with different complications. Studies on BD have shown that different inflammatory cytokine profiles were present in different BD subtypes, such as dominant eye involvement, and vascular or neurological involvement [45]. It is possible that Alu-dependent inflammatory pathways play a role in a certain subset of BD profiles, but not in others. It should be noted that the sample size in this study was rather limited and the difference was marginal. Further analysis in larger patient groups, as well as studies on different patient profiles, will be useful to delineate whether Alu hypomethylation contributes to BD pathogenesis.

## 5. Conclusions

In conclusion, we demonstrated that there is an increase in the frequency of unmethylated (^u^C^u^C) Alu alleles in PBMCs and neutrophils of inactive BD patients, while there was no significant difference in terms of hypomethylation between active BD samples and controls. This is contrary to the findings for autoimmune diseases such as SLE, RA, and psoriasis, in which LINE-1, but not Alu, is significantly hypomethylated in patients compared with healthy controls. This result might be explained in part by the different pathogenesis between BD, which is more similar to the autoinflammatory disease, and other classic autoimmune diseases. Further study of the epigenetic alterations, and the role and regulation of Alu transcripts in BD and its clinical significance, should be pursued to gain a better understanding of the disease. Such research could lead to the discovery of uncharacterized mechanisms that could be useful as diagnostic biomarkers and therapeutic targets.

## Figures and Tables

**Table 1:** Patient demographics and medications.

	Group	Age	Sex	Disease score	Medications	Active extraocular findings	Total extraocular findings	Active/inactive uveitis
1	Inactive	32	M	0	Interferon Alpha 2a (3 million) every other day	—	OA, GU, folliculitis, pathergy positivity, and joint involvement	Inactive
2	Inactive	25	F	0	Cyclosporine-A 150 mg/day and Azathioprine 100 mg	—	OA, GU, and EN	Inactive
4	Inactive	27	M	0	Infliximab infusions (5 mg /kg) at 0, 2, and 6 and then every 4–8 weeks was administered	—	OA, GU, and folliculitis	Inactive
5	Inactive	41	M	0	Cyclosporine 2.5 mg/kg/day, Azathioprine 100 mg/day	—	OA, GU, EN, and joint involvement	Inactive
6	Inactive	37	F	0	Interferon Alpha 2a (3 million) every other day	—	OA, GU, EN, folliculitis, pathergy positivity, and joint involvement	Inactive
7	Inactive	33	M	0	Azathioprine 100 mg/day	—	OA, GU, folliculitis, pathergy positivity, joint involvement, vascular involvement, and neurological involvement	Inactive
8	Inactive	41	M	0	Cyclosporine-A 2.5 mg/kg/day, Azathioprine 100 mg/day	—	OA, folliculitis, and pathergy positivity	Inactive
9	Inactive	40	F	0	Unmedicated	—	OA, GU, folliculitis, and pathergy positivity	Inactive
10	Inactive	57	M	0	Unmedicated	—	OA, GU, EN, joint involvement, and vascular involvement	Inactive
11	Inactive	26	M	0	Cyclosporine-A 2.5 mg/kg/day, Azathioprine 100 mg/day	—	OA, GU, EN, folliculitis, and joint involvement	Inactive
12	Inactive	21	F	3	Unmedicated	OA	OA, GU, EN, and folliculitis	Inactive
13	Inactive	46	M	0	Cyclosporine-A 2.5 mg/kg/day, Azathioprine 100 mg/day	—	OA, GU	Inactive
16	Inactive	34	F	0	Unmedicated	—	OA, GU, EN, pathergy positivity, and joint involvement	Inactive
17	Inactive	54	M	0	Azathioprine 100 mg/day	—	OA, GU, EN, folliculitis, joint involvement, vascular involvement, and neurological involvement	Inactive
18	Inactive	40	M	0	Cyclosporine-A 2.5 mg/kg/day, Azathioprine 100 mg/day	—	OA, GU, EN, and joint involvement	Inactive
19	Inactive	35	M	0	Interferon Alpha 2a (3 million) every other day	—	OA, GU, and pathergy positivity	Inactive
20	Inactive	36	M	0	Interferon Alpha 2a (3 million) every other day	—	OA, GU, EN, pathergy positivity, joint involvement, and vascular involvement	Inactive

1	Active	46	M	7	Interferon Alpha 2a (3 million) every other day	OA, GE	OA, GU, EN, pathergy positivity, and vascular involvement	Active U
2	Active	33	M	8	Interferon Alpha 2a (3 million) every other day	OA, GU, EN	OA, GU, pathergy positivity, EN, and joint involvement	Active
3	Active	21	M	7	Interferon Alpha 2a (3 million) every other day	OA, PP	OA, GU, EN, pathergy positivity, and joint involvement	Active
4	Active	31	M	7	Cyclosporine-A 2.5 mg/kg/day, Azathioprine 100 mg/day	OA, PP	OA, GU, folliculitis, and pathergy positivity	Active
5	Active	30	M	7	Interferon Alpha 2a (3 million) every other day	OA, PP	OA, GU, EN, folliculitis, pathergy positivity, joint involvement, and neurological involvement	Active
6	Active	37	F	7	Interferon Alpha 2a (3 million) every other day	OA, PP	OA, GU, EN, folliculitis, and joint involvement,	Active
7	Active	49	F	5	Cyclosporine-A 2.5 mg/kg/day, Azathioprine 100 mg/day	OA	OA, GU, EN, folliculitis, pathergy positivity, and joint involvement	Active
8	Active	18	F	5	Interferon Alpha 2a (3 million) every other day	OA	OA, GU, and EN	Active
9	Active	31	M	7	Interferon alpha 2a (3 Million) every other day	OA, PP	OA, GU, folliculitis, pathology positivity, and vascular involvement	Active
10	Active	34	M	5	Cyclosporine-A 2.5 mg/kg/day, Azathioprine 100 mg/day	OA	OA, PP, and Joint involvement	Active U
11	Active	27	F	7	Interferon Alpha 2a (3 million) every other day	OA, GU	OA, GU	Active U
12	Active	38	M	9	Cyclosporine-A 2.5 mg/kg/day, Azathioprine 100 mg/day	OA	OA, GU, folliculitis, and GI	Active U

F: female; M: male

U: Uveitis; OA: oral ulcers; GU: genital ulcers; EN: erythema nodosum; GI: gastrointestinal involv.

**Table 2:** LINE-1 methylation frequency of BD patients and healthy controls. The results represented as the average ± standard deviation.

	% ^m^C	% ^m^C^m^C	% ^u^C^m^C	% ^m^C^u^C	% ^u^C^u^C	% ^u^C^m^C + ^m^C^u^C
PBMC control (*n* = 15)	81.76 ± 2.04	81.76 ± 2.04	9.62 ± 3.39	16.42 ± 1.08	5.22 ± 1.07	26.05 ± 3.87
PBMC active (*n* = 12)	81.61 ± 1.63	68.93 ± 2.59	8.29 ± 1.92	17.07 ± 1.39	5.71 ± 0.80	25.37 ± 2.03
PBMC inactive (*n* = 17)	81.15 ± 1.18	68.21 ± 1.57	9.80 ± 1.97	16.07 ± 1.28	5.92 ± 1.59	25.87 ± 2.10
PBMC all patients (*n* = 29)	81.34 ± 1.37	68.51 ± 2.04	9.18 ± 2.06	16.48 ± 1.40	5.83 ± 1.31	25.66 ± 2.05

Neutrophil control (*n* = 15)	81.06 ± 1.94	67.73 ± 2.92	11.01 ± 2.03	15.64 ± 0.55	5.61 ± 1.20	26.65 ± 2.23
Neutrophil active (*n* = 12)	82.07 ± 0.96	69.12 ± 1.39	10.06 ± 1.20	15.86 ± 1.32	4.97 ± 0.61	25.92 ± 0.98
Neutrophil inactive (*n* = 17)	81.35 ± 1.25	67.82 ± 2.04	11.06 ± 2.54	16.01 ± 1.73	5.11 ± 0.89	27.07 ± 1.91
Neutrophil all patients (*n* = 29)	81.65 ± 1.18	68.36 ± 1.89	10.64 ± 2.12	15.95 ± 1.55	5.05 ± 0.78	26.59 ± 1.67

**Table 3:** Alu methylation frequency of BD patients and healthy controls. The results are represented as the average ± standard deviation.

	% ^m^C	% ^m^C^m^C	% ^u^C^m^C	% ^m^C^u^C	% ^u^C^u^C	% ^u^C^m^C + ^m^C^u^C
PBMC control (*n* = 15)	42.14 ± 2.07	14.06 ± 2.96	31.90 ± 2.28	24.25 ± 1.08	29.79 ± 2.08	56.15 ± 2.99
PBMC active (*n* = 12)	41.57 ± 0.92	13.72 ± 1.21	31.25 ± 0.93	24.46 ± 0.76	30.58 ± 1.13	55.71 ± 1.45
PBMC inactive (*n* = 17)	41.50 ± 1.03	14.13 ± 1.51	30.62 ± 0.94	24.11 ± 0.82	31.13 ± 1.11^a^	54.73 ± 1.66
PBMC all patients (*n* = 29)	41.53 ± 0.97	13.96 ± 1.39	30.88 ± 0.97	24.26 ± 0.80	30.90 ± 1.13^b^	55.14 ± 1.62

Neutrophil control (*n* = 15)	42.88 ± 2.19	14.25 ± 3.68	32.39 ± 1.81	24.87 ± 1.40	28.49 ± 1.03	57.26 ± 3.16
Neutrophil active (*n* = 12)	42.89 ± 0.40	15.17 ± 1.58	30.83 ± 2.60	24.60 ± 0.83	29.40 ± 1.64	55.43 ± 3.11
Neutrophil inactive (*n* = 17)	42.43 ± 1.52	14.91 ± 1.88	30.88 ± 0.97	24.15 ± 0.69	30.06 ± 1.49^c^	55.03 ± 1.50^e^
Neutrophil all patients (*n* = 29)	42.62 ± 1.20	15.02 ± 1.73	30.86 ± 1.79	24.34 ± 0.77	29.78 ± 1.56^d^	55.20 ± 2.26^f^

a, *p* = 0.03 for the frequency of the unmethylated allele (^u^C^u^C) in PBMCs in inactive BD patients versus healthy controls.

b, *p* = 0.03 for the frequency of the unmethylated allele (^u^C^u^C) in PBMCs in BD patients versus healthy controls.

c, *p* = 0.002 for the frequency of the unmethylated allele (^u^C^u^C) in neutrophils in inactive BD patients versus healthy controls.

d, *p* = 0.006 for the frequency of the unmethylated allele (^u^C^u^C) in neutrophils in BD patients versus healthy controls.

e, *p* = 0.02 for the frequency of the partially methylated allele (^u^C^m^C + ^m^C^u^C) in neutrophils in inactive BD patients versus healthy controls.

f, *p* = 0.055 for the frequency of the partially methylated allele (^u^C^m^C + ^m^C^u^C) in neutrophils in BD patients versus healthy control.

## References

[1] Behçet H. (1937). Über rezidivierende, aphthöse durch ein virus verursachte geschwure am mund, am auge und an den genitalen. *Dermatologische Wochenschrift*.

[2] Düzgün N., Ayaşlioğlu E., Tutkak H., Aydintuğ O. T. (2005). Cytokine inhibitors: soluble tumor necrosis factor receptor 1 and interleukin-1 receptor antagonist in Behçet's disease. *Rheumatology International*.

[3] Mege J.-L., Dilsen N., Sanguedolce V. (1993). Overproduction of monocyte derived tumor necrosis factor *α*, interleukin (IL) 6, IL-8 and increased neutrophil superoxide generation in Behcet's disease. A comparative study with familial Mediterranean fever and healthy subjects. *Journal of Rheumatology*.

[4] Eksioglu-Demiralp E., Direskeneli H., Kibaroglu A., Yavuz S., Ergun T., Akoglu T. (2001). Neutrophil activation in Behçet's disease. *Clinical and Experimental Rheumatology*.

[5] Gül A. (2005). Behçet's disease as an autoinflammatory disorder. *Current Drug Targets: Inflammation and Allergy*.

[6] Atagunduz P., Ergun T., Direskeneli H. (2003). MEFV mutations are increased in Behçet's disease (BD) and are associated with vascular involvement. *Clinical and Experimental Rheumatology*.

[7] Yazici H., Tuzun Y., Pazarli H. (1984). Influence of age of onset and patient's sex on the prevalence and severity of manifestations of Behcet's syndrome. *Annals of the Rheumatic Diseases*.

[8] Tursen U., Gurler A., Boyvat A. (2003). Evaluation of clinical findings according to sex in 2313 Turkish patients with Behçet's disease. *International Journal of Dermatology*.

[9] Yurdakul S., Yazici H. (2008). Behçet's syndrome. *Best Practice and Research: Clinical Rheumatology*.

[10] Direskeneli H. (2006). Autoimmunity vs autoinflammation in Behcet's disease: do we oversimplify a complex disorder?. *Rheumatology*.

[11] Koné-Paut I., Geisler I., Wechsler B. (1999). Familial aggregation in Behcet's disease: high frequency in siblings and parents of pediatric probands. *The Journal of Pediatrics*.

[12] Gül A., Ohno S., Yazıcı Y., Yazıcı H. (2010). Genetics of Behçet's disease. *Behçet's Syndrome*.

[13] Verity D. H., Marr J. E., Ohno S., Wallace G. R., Stanford M. R. (1999). Behcet's disease, the Silk Road and HLA-B51: historical and geographical perspectives. *Tissue Antigens*.

[14] Gül A. (2001). Behçet's disease: an update on the pathogenesis. *Clinical and Experimental Rheumatology*.

[15] Remmers E. F., Cosan F., Kirino Y. (2010). Genome-wide association study identifies variants in the MHC class I, IL10, and IL23R-IL12RB2 regions associated with Behçet's disease. *Nature Genetics*.

[16] Mizuki N., Meguro A., Ota M. (2010). Genome-wide association studies identify IL23R-IL12RB2 and IL10 as Behçet's disease susceptibility loci. *Nature Genetics*.

[17] Kirino Y., Bertsias G., Ishigatsubo Y. (2013). Genome-wide association analysis identifies new susceptibility loci for Behçet's disease and epistasis between HLA-B^∗^51 and ERAP1. *Nature Genetics*.

[18] Ahn J. K., Cha H.-S., Lee J., Jeon C. H., Koh E.-M. (2012). Correlation of DEFA1 gene copy number variation with intestinal involvement in Behcet's disease. *Journal of Korean Medical Science*.

[19] Yu H., Luo L., Wu L. (2015). FAS gene copy numbers are associated with susceptibility to Behçet disease and VKH syndrome in Han Chinese. *Human Mutation*.

[20] Zheng M., Yu H., Zhang L. (2015). Association of ATG5 Gene polymorphisms with Behcet's disease and ATG10 gene polymorphisms with VKH syndrome in a Chinese Han population. *Investigative Opthalmology & Visual Science*.

[21] Qi J., Hou S., Zhang Q. (2013). A functional variant of pre-miRNA-196a2 confers risk for Behcet's disease but not for Vogt-Koyanagi-Harada syndrome or AAU in ankylosing spondylitis. *Human Genetics*.

[22] Yu H., Liu Y., Bai L., Kijlstra A., Yang P. (2014). Predisposition to Behçet's disease and VKH syndrome by genetic variants of miR-182. *Journal of Molecular Medicine*.

[23] Zhou Q., Hou S., Liang L. (2014). MicroRNA-146a and Ets-1 gene polymorphisms in ocular Behçet's disease and Vogt-Koyanagi-Harada syndrome. *Annals of the Rheumatic Diseases*.

[24] Hughes T., Ture-Ozdemir F., Alibaz-Oner F., Coit P., Direskeneli H., Sawalha A. H. (2014). Epigenome-wide scan identifies a treatment-responsive pattern of altered dna methylation among cytoskeletal remodeling genes in monocytes and CD4+ T cells from patients with behçet's disease. *Arthritis and Rheumatology*.

[25] Levy S., Sutton G., Ng P. C. (2007). The diploid genome sequence of an individual human. *PLoS Biology*.

[26] Bannert N., Kurth R. (2004). Retroelements and the human genome: new perspectives on an old relation. *Proceedings of the National Academy of Sciences of the United States of America*.

[27] Zhang Z., Zhang R. (2015). Epigenetics in autoimmune diseases: pathogenesis and prospects for therapy. *Autoimmunity Reviews*.

[28] Nakkuntod J., Avihingsanon Y., Mutirangura A., Hirankarn N. (2011). Hypomethylation of LINE-1 but not Alu in lymphocyte subsets of systemic lupus erythematosus patients. *Clinica Chimica Acta*.

[29] Sukapan P., Promnarate P., Avihingsanon Y., Mutirangura A., Hirankarn N. (2014). Types of DNA methylation status of the interspersed repetitive sequences for LINE-1, Alu, HERV-E and HERV-K in the neutrophils from systemic lupus erythematosus patients and healthy controls. *Journal of Human Genetics*.

[30] Yooyongsatit S., Ruchusatsawat K., Noppakun N., Hirankarn N., Mutirangura A., Wongpiyabovorn J. (2015). Patterns and functional roles of LINE-1 and Alu methylation in the keratinocyte from patients with psoriasis vulgaris. *Journal of Human Genetics*.

[31] Ali M., Veale D. J., Reece R. J. (2003). Overexpression of transcripts containing LINE-1 in the synovia of patients with rheumatoid arthritis. *Annals of the Rheumatic Diseases*.

[32] Karouzakis E., Gay R. E., Michel B. A., Gay S., Neidhart M. (2009). DNA hypomethylation in rheumatoid arthritis synovial fibroblasts. *Arthritis and Rheumatism*.

[33] Miousse I. R., Koturbash I. (2015). The fine LINE: methylation drawing the cancer landscape. *BioMed Research International*.

[34] Luo Y., Lu X., Xie H. (2014). Dynamic Alu methylation during normal development, aging, and tumorigenesis. *BioMed Research International*.

[35] Garaud S., Le Dantec C., Jousse-Joulin S. (2009). IL-6 modulates CD5 expression in B cells from patients with lupus by regulating DNA methylation. *The Journal of Immunology*.

[36] Garaud S., Le Dantec C., Berthou C., Lydyard P. M., Youinou P., Renaudineau Y. (2008). Selection of the alternative exon 1 from the cd5 gene down-regulates membrane level of the protein in B lymphocytes. *Journal of Immunology*.

[37] Sicat J., Sutkowski N., Huber B. T. (2005). Expression of human endogenous retrovirus HERV-K18 superantigen is elevated in juvenile rheumatoid arthritis. *Journal of Rheumatology*.

[38] Magnusson M., Magnusson S., Vallin H., Rönnblom L., Alm G. V. (2001). Importance of CpG dinucleotides in activation of natural IFN-*α*-producing cells by a lupus-related oligodeoxynucleotide. *Scandinavian Journal of Immunology*.

[39] Crow M. K. (2010). Long interspersed nuclear elements (LINE-1): potential triggers of systemic autoimmune disease. *Autoimmunity*.

[40] Jintaridth P., Mutirangura A. (2010). Distinctive patterns of age-dependent hypomethylation in interspersed repetitive sequences. *Physiological Genomics*.

[41] Bollati V., Schwartz J., Wright R. (2009). Decline in genomic DNA methylation through aging in a cohort of elderly subjects. *Mechanisms of Ageing and Development*.

[42] Baba Y., Murata A., Watanabe M., Baba H. (2014). Clinical implications of the LINE-1 methylation levels in patients with gastrointestinal cancer. *Surgery Today*.

[43] Tarallo V., Hirano Y., Gelfand B. D. (2012). DICER1 loss and Alu RNA induce age-related macular degeneration via the NLRP3 inflammasome and MyD88. *Cell*.

[44] Gelfand B. D., Wright C. B., Kim Y. (2015). Iron toxicity in the retina requires Alu RNA and the NLRP3 inflammasome. *Cell Reports*.

[45] Yuksel S., Eren E., Hatemi G. (2014). Novel NLRP3/cryopyrin mutations and pro-inflammatory cytokine profiles in Behcet's syndrome patients. *International Immunology*.

